# Hormonal Orchestration of Bud Dormancy Cycle in Deciduous Woody Perennials

**DOI:** 10.3389/fpls.2019.01136

**Published:** 2019-09-18

**Authors:** Jianyang Liu, Sherif M. Sherif

**Affiliations:** Alson H. Smith Jr. Agricultural Research and Extension Center, School of Plant and Environmental Sciences, Virginia Tech, Winchester, VA, United States

**Keywords:** phytohormones, woody species, bud dormancy, endodormancy, DAM genes, hormone signaling

## Abstract

Woody perennials enter seasonal dormancy to avoid unfavorable environmental conditions. Plant hormones are the critical mediators regulating this complex process, which is subject to the influence of many internal and external factors. Over the last two decades, our knowledge of hormone-mediated dormancy has increased considerably, primarily due to advancements in molecular biology, omics, and bioinformatics. These advancements have enabled the elucidation of several aspects of hormonal regulation associated with bud dormancy in various deciduous tree species. Plant hormones interact with each other extensively in a context-dependent manner. The dormancy-associated MADS (DAM) transcription factors appear to enable hormones and other internal signals associated with the transition between different phases of bud dormancy. These proteins likely hold a great potential in deciphering the underlying mechanisms of dormancy initiation, maintenance, and release. In this review, a recent understanding of the roles of plant hormones, their cross talks, and their potential interactions with DAM proteins during dormancy is discussed.

## Introduction

Bud dormancy is an essential adaptation, which allows temperate woody perennials to survive adverse environmental conditions during winter. During dormancy, plants experience arrested growth and reduced metabolic activities (Arora et al., 2003). According to the source of the signals that induce dormancy, dormancy can be categorized into three classes: paradormancy (PD), endodormancy (ED), and ecodormancy (ECD) (Lang, 1987). In PD, lateral bud growth is suppressed by the terminal bud, a phenomenon known as apical dominance. In ED, short days (SDs) and/or low temperatures trigger internal bud signals, which enable buds to become tolerant to temperatures well below freezing. Endodormant buds track chilling units and will not resume growth until the fulfillment of the chilling requirement. ECD marks the last stage of dormancy where buds resume the ability to grow but are inhibited by unfavorable weather conditions. While this classification allows convenient references to the different stages of the dormancy–growth cycle of deciduous perennials, the nomenclature of PD and ECD has raised some concerns and confusions as they lack many genetic and biological hallmarks that are characteristic of a true dormancy. At the core of a true dormancy is the “inability to resume growth from meristems under favorable conditions” (Rohde and Bhalerao, 2007), which apparently does not apply to PD or ECD. In view of this, several authors have advocated the prudent use of dormancy, especially in the discussion of dormancy mechanisms at the molecular or cellular levels (Rohde and Bhalerao, 2007; Cooke et al., 2012; Considine and Considine, 2016). In this review, we refer to ED as dormancy unless specified otherwise and PD or ECD *per se*. Dormancy is a highly regulated and complex process and is subjected to the influences of many internal and external factors. Plant hormones have been shown to be the most significant internal mediators in the control of dormancy cycle in deciduous trees.

Plant hormones, or phytohormones, are naturally occurring small signaling molecules that affect plant physiological metabolism at low concentrations (Davies, 2010). Plant hormones regulate developmental and growth processes throughout the plant’s life cycle and also trigger adaptive responses induced by external stimuli such as environmental changes and biotic or abiotic stresses. Conventional plant hormones include five major groups: abscisic acid (ABA), gibberellin (GA), ethylene (ET), auxin (indole-3-acetic acid, IAA), and cytokinin (CK). Other major plant-produced substances known to exert hormone-like functions in plants include jasmonates (JAs), brassinosteroids (BRs), strigolactones (SL), and salicylic acid (SA). Many of these plant hormones have been found to participate in the highly complex orchestration of bud dormancy. Thus far, our knowledge in deciphering the mechanisms by which dormancy is regulated by plant hormones still remains limited. However, several breakthroughs associated with mechanistic aspects of hormone signaling at the molecular and subcellular levels have elucidated hormone perception, signal transduction, and signal interplay in several major hormones (Chow and Mccourt, 2006; Santner and Estelle, 2009; Santner et al., 2009). Genetic mutagenesis in model plant species have also allowed us to examine the function of a different element of hormone biosynthesis and signaling pathways under various conditions, including dormancy. This, along with the recent advances in omics and bioinformatics that provide global views of all relevant genetic events at a specific time, has considerably advanced our knowledge of hormone regulation as it applies to bud dormancy. Such quick gains in research, knowledge, and technologies necessitate a summarization of recent findings and suggestions for future research. A comprehensive overview of the underlying molecular and biochemical mechanisms involved in bud dormancy will also help researchers design practical approaches to address critical issues in agriculture, horticulture, and forestry, such as global warming and spring frost. In this review, we focus on topics related to recent findings associated with bud dormancy, with an emphasis on the functions and interactions of plant hormones during bud dormancy, particularly in deciduous fruit trees.

## Abscisic Acid

Plant hormone ABA regulates a great number of aspects in plant growth and development and is also an important messenger of stress responses (Finkelstein, 2013). The primary role of ABA in plants is to repress growth and to promote organ senescence and abscission (Finkelstein, 2013; Zheng et al., 2015). In light of this, ABA is of particular importance in regulating dormancy, since dormancy in essence is the suspension of meristematic growth and successful dormancy establishment entails cessation of the overall plant growth (Cooke et al., 2012). The central role of ABA in regulating bud dormancy has been extensively documented in many physiological, genetic, and molecular studies.

### Endogenous ABA Changes During Dormancy

It has been widely observed that endogenous ABA levels increase at dormancy establishment and decrease towards dormancy release (transition from ED to ECD). For example, ABA content in grapevine (*Vitis vinifera*) buds increases up to threefold at the onset of dormancy and then decreases gradually towards the release of dormancy, indicated by the increasing bud break rate from node cuttings in forcing conditions (Zheng et al., 2015). Similar results were also observed in some other woody species including peach (*Prunus persica*) (Wang et al., 2015), pear (*Pyrus pyrifolia*) (Tuan et al., 2017; Li et al., 2018), and sweet cherry (*Prunus avium*) (Chmielewski et al., 2018). It has been proposed that the increase of ABA content is triggered by SD photoperiod prior to the establishment of dormancy (Ruttink et al., 2007) and its decline is concomitant with chilling accumulation (Li et al., 2018). Such parallel relationship between ABA levels and the depth of dormancy suggests ABA’s role in the initiation and progression of the dormancy cycle and that ABA may also be involved in mediating environmental signals.

The importance of ABA in dormancy regulation is also evidenced by the precocious release of dormancy when ABA content in dormant buds is artificially reduced. Early study in rose (*Rosa hybrida*) indicated that application of ABA synthesis inhibitor fluridone to dormant buds initiated the growth of new leaf primordia (Le Bris et al., 1999), which led the authors to suggest that continued *in situ* ABA synthesis is required for the maintenance of bud dormancy. Furthermore, ABA catabolism in grapevine was effectively activated, and its content was reduced after treatment of buds with hydrogen cyanamide (HC), an efficient bud-breaking chemical (Ophir et al., 2009; Zheng et al., 2015). These findings are also supported by a transgenic study in pear (*P. pyrifolia*), in which dormancy release was accelerated when an ABA catabolism enzyme 8′-hydroxylase (*ABA8ʹOH*) was overexpressed (Li et al., 2018). These studies support the notion that ABA is an effective suppressor of primordia growth during dormancy and even though ABA is on the decline after dormancy has been established, basal levels of ABA and *de novo* ABA production may still be required to keep the buds at dormancy, and the continuous reduction of ABAs contributes to the release of dormancy.

### Exogenous ABA Effect on Bud Dormancy

Studies of exogenous ABA application have provided more insight into how ABA affects dormancy. In several woody species, exogenous ABA application was found to promote dormancy initiation and to delay bud break (Dutcher and Powell, 1972; Mielke and Dennis, 1978; Lionakis and Schwabe, 1984; Li et al., 2018). However, closer examination shows that the inhibitory effect of exogenous ABA on bud cuttings diminishes as dormancy intensifies and disappears after the dormancy is released. ABA inhibitory effect on bud break is also affected by chilling accumulation, as injection of ABA only inhibited bud break of Japanese pear (*P*yrus *fauriei*) shoots that were exposed to 200–600 chilling hours, but not those exposed to 800–1,200 chilling hours (Tamura et al., 2002). Together, this proposes that a non-ABA-mediated regulatory mechanism, which controls the dormancy transition at certain phases or when given requirements have been met, may exist. This can be supported by the observation that the variation of ABA levels is not always in exact synchronization with dormancy progression. For instance, while the decrease in ABA levels was found to commence somewhat prior to the release of dormancy (Or et al., 2000; Zhang et al., 2018), Chmielewski et al. (2018) reported that ABA levels in sweet cherry did not decline until buds have entered ECD. Thus, it appears that even though ABA is a key regulator of dormancy, some separate regulatory networks, which respond more closely to environmental stimuli, may still be at play and override ABA’s regulatory action at certain stages.

In contrast to applications of exogenous ABA to cuttings, effects of ABA applied on whole plants produced inconsistent results, with application timing appearing to be critical. Foliar application of ABA to dormant peach trees prior to budburst slightly accelerated bloom progression (Parker et al., 2012). Similarly, spring application of ABA on field-grown grapevine produced little or no inhibitory effect on budburst (Hellman et al., 2006). On the other hand, fall application of ABA on nursery apple trees (*Malus domestica*) promoted the occurrence of the physiological events preceding dormancy commencement such as N mobilization from leaves to stems, cold acclimation in stems, and shoot growth cessation (Guak and Fuchigami, 2015). ABA application on grapevines was more effective in inducing deeper dormancy during early autumn between the veraison and post veraison stages compared to mid-autumn applications (Li and Dami, 2016). Furthermore, leaf age was also found to influence the effectiveness of exogenous ABA, as grapevines with older leaves were more responsive to exogenous ABA in inducing dormancy compared to those with younger leaves (Zhang et al., 2011). Plant responses to exogenous ABA depend on the successful penetration of ABA into tissue (Addicott and Lyon, 1969). This may explain why cuttings are more consistently responsive to ABA applications compared to whole-plant applications, as cuttings are usually incubated with ABA solution for an extended period of time to facilitate cuticular penetration of ABA. In contrast, uptake of ABA by whole-plant application is more dependent on successful cuticular penetration, frequency of applications, and several environmental variables, which may all contribute to the inconsistency of ABA effect on whole-plant application.

### ABA Metabolism and Bud Dormancy

In higher plants, ABA metabolism is finely coordinated to ensure proper growth and effective stress responses. The ABA biosynthesis pathway has been reviewed extensively (Marin et al., 1996; Milborrow, 2001; Finkelstein, 2013; Liao et al., 2018). The first step of ABA production is the epoxidation of zeaxanthin to antheraxanthin by zeaxanthin epoxidase (ZEP). Next, antheraxanthin is converted to neoxanthin or violaxanthin, which are both cleaved to form xanthoxin by 9-*cis*-epoxycarotenoid dioxygenase (NCED) (Liotenberg et al., 1999; Taylor et al., 2000). In the following two steps, xanthoxin is first dehydrated by alcohol dehydrogenase and then oxidized to ABA by aldehyde oxidase (Bittner et al., 2001; Cheng et al., 2002). Though ZEP has been shown to affect ABA biosynthesis in some species (Nambara and Marion-Poll, 2005), severe *zep* mutation could not completely eliminate the production of ABA, suggesting the existence of a ZEP-independent minor pathway for ABA biosynthesis (Barrero et al., 2005). In contrast, the NCED-mediated violaxanthin cleavage is a rate-limiting and committed step, constituting a regulation pivot in controlling ABA biosynthesis, and has received substantial attention in the ABA-related studies (Nambara and Marion-Poll, 2005).

Accumulating evidence has indicated that ABA biosynthesis is involved in controlling dormancy. Upregulation of ABA synthetic enzyme *NCED* at the onset of dormancy and its downregulation during dormancy release were observed in many species such as peach (Wang et al., 2015), pear (*P. pyrifolia*) (Li et al., 2018), and grapevine (Zheng et al., 2015), to name a few. However, it was noted that various *NCED* homologs follow distinct expression patterns in these plant species during dormancy. For example, *NCED1* in peach is expressed more in vegetative buds than in floral buds, while *NCED2* has higher expression in floral buds (Wang et al., 2015). In pear, *NCED2* and *NCED3* are highly expressed at the initiation of dormancy, but *NCED1* peaks only towards the dormancy release (Li et al., 2018). Among the three homologs of *NCED* genes in grapevine, only *NCED1* was detectable during dormancy (Zheng et al., 2015). These findings indicate the existence of a complex regulatory network of ABA biosynthesis, in which *NCED* genes are probably regulated by relatively independent mechanisms and are expressed in an organ-specific manner.

In catabolism, ABA is primarily degraded through the 8′ position hydroxylation by enzyme ABA8′OH, a cytochrome P450 monooxygenase, encoded by the *CYP707A* gene family (Kushiro et al., 2004; Umezawa et al., 2006). ABA8′OH is a key regulator of ABA catabolism, and mutation of *CYP707A* can lead to accumulation of high levels of ABA (Finkelstein, 2013). ABA hydroxylation produces two major catabolites: phaseic acid (PA) and dihydrophaseic acid (DPA), both of which are commonly used to indicate the level of ABA catabolism (Destefano-Beltran et al., 2006). Recent studies indicate that ABA8′OH is involved in the control of dormancy release, and its synthesis is regulated at the transcription level. In grapevine, transcript levels of *VvCYP707A4*, an *ABA8′OH* gene, drastically increased about 20 days before the onset of dormancy release and remained steady throughout the dormancy release period (Zheng et al., 2015). The upregulation of *VvCYP707A4* was concomitant with the accumulation of ABA catabolites, such as neoPA, PA, and DPA. Similarly, *PpCYP707A2* and *PpCYP707A3* in peach and *CYP707A* in Japanese pear were all found to have heightened expression during the release of dormancy (Wang et al., 2015; Tuan et al., 2017; Li et al., 2018). Li et al. (2018) noted that an increase of *PpCYP707A3* in flower buds of white pear (*P. pyrifolia*) during dormancy was related to chilling accumulation, further confirming the link between ABA and the fulfillment of chilling requirements.

Homeostasis of ABA in plants is essential for normal growth and development, in which buds are both the target site for ABA to act upon and the principal location of ABA metabolism and catabolism. Transcription studies indicate that the alteration of one process is often accompanied by an opposite change in the other process, suggesting that these two processes are closely co-regulated. For example, *VvNCED* expression in grape peaks during early dormancy while expression of *VvCYC707A4* remains very low. In contrast, during late dormancy, *VvNCED* expression starts to decline as *VvCYC707A4* rapidly increases (Zheng et al., 2015). Furthermore, Li et al. (2018) found that *PpCYP707A1-5* expression was drastically upregulated in ABA-treated pear (*P. pyrifolia*) trees while *PpNCED* expression remained unaltered. This observation suggests that ABA may stimulate its own degradation *via* a negative feedback pathway. In contrast, positive feedback of ABA regulation has been observed in *Arabidopsis* where ABA synthesis genes, including *NCED*, were induced by exogenous ABA (Cheng et al., 2002; Xiong and Zhu, 2003). This mechanism seems to function only under certain genetic backgrounds (such as *aba* mutants) and stress conditions (such as drought) (Xiong et al., 2002). As a plant growth regulator, ABA can be used in agricultural practices to manipulate dormancy release and bloom date, thus saving early-bloom varieties and species from potential spring freezes. However, the systems by which endogenous ABA and its signal pathway are affected by exogenous ABA during dormancy need further elucidation.

ABA deactivation can also be achieved through conjugation. In this process, an ABA molecule typically conjugates with glucosyl ester to form ABA glucosyl ester (ABA-GE) by the enzyme glucosyltransferase (Piotrowska and Bajguz, 2011). ABA-GE lacks a direct biological function and is generally believed to serve as the storage form of ABA, which can be relocated and disrupted to release ABA in response to stresses, such as dehydration (Sauter et al., 2002; Lee et al., 2006; Xu et al., 2012). A recent study has indicated that the glucosyl ester may also act as an ABA antagonist by regulating ABA supply during bud dormancy. In sweet cherry buds, ABA-GE levels were lower than ABA levels during dormancy but increased as buds transitioned from dormancy to ECD (Chmielewski et al., 2018). Similarly, a homolog of glucosyltransferase was found to be highly expressed in *Prunus mume* during later stages of dormancy (Zhang et al., 2018). While these reports confirm the role of ABA glucosylation in ABA deactivation during dormancy release, it still remains unclear to what extent this mechanism supplements the oxidative catabolism of ABA and how it is spatially and temporally regulated.

### ABA Signaling Pathway and Dormancy

In addition to ABA metabolism, ABA responses are also regulated at the signal transduction level. The ABA signaling pathway consists of three key components: ABA receptors, protein phosphate 2Cs (PP2Cs), and SNF1-related protein kinase 2s (SnRK2s). The three types of high-affinity ABA-binding proteins that have been recently identified are regulatory pyrabactin resistance (PYR), pyrabactin like (PYL), and components of ABA receptors 1 (RCAR) (Raghavendra et al., 2010). ABA forms a complex with PP2Cs after binding to the RCAR/PYR/PYL receptor and thus deactivates PP2Cs, which suppress the activity of SnRK2s through dephosphorylation. Phosphorylated SnRK2s activate the ABA-responsive element (ABRE)-binding protein/ABRE-binding factors (AREB/ABF) transcription factors, which subsequently trigger the downstream ABA responses by binding to the ABREs (Soon et al., 2012).

Genetic studies have indicated that transcript levels of these central components of ABA signaling pathway are modulated by environmental signals and levels of ABA as well. In grapevine, some *RCAR* and *PP2C* genes are highly induced by cold, drought, salt, and exogenous ABA, and their induction differs greatly across organs (Boneh et al., 2012). In a transcriptomic study with pear, Li et al. (2018) showed that the expression of ABA receptor *PYL* and the positive regulator *SnRK2s* was upregulated at the entrance of dormancy; and in contrast, the expression of *PP2Cs*, the suppressor of ABA signaling, remained low during dormancy and increased as ABA content decreased towards the end of the dormancy. Furthermore, exogenous ABA promoted the expression of *SnRK2s* but suppressed that of *PP2Cs*. A similar result was also obtained in Japanese pear, in which *PP2C* genes were upregulated and *SnRK2s* downregulated after buds exited from dormancy (Bai et al., 2013). Ruttink et al. (2007) indicated that changes of ABA signaling component expression were induced in response to the SD photoperiod. Taken together, these results suggest that the ABA signaling pathway is subject to the influence of both seasonal variation and ABA contents during dormancy.

Early studies in *Arabidopsis* have demonstrated that transcriptional factors from the ABA-insensitive (ABI) group can individually or collaboratively mediate the expression of ABA-inducible genes (Nakamura et al., 2001). Among them, ABI3 is believed to play an important role in seed embryo maturation and dormancy by positively regulating the ABA signaling pathway (Nambara et al., 1995; Finkelstein et al., 2008). In poplar (*Populus trichocarpa*), ABI3 was found to be expressed in the embryonic leaves inside the bud during bud set, and the overexpression or silencing of *ABI3* caused alterations in bud development, indicating its crucial role in bud formation (Rohde et al., 2002). Broader functions of ABI3 were also revealed in vegetative tissue, in which ABI3 regulates plastid differentiation (Rohde et al., 2000) and induces ABA responses through targeting the *ABREs* (Nakashima et al., 2006).

### ABA and Plasmodesmata Controls

**Figure 1:**
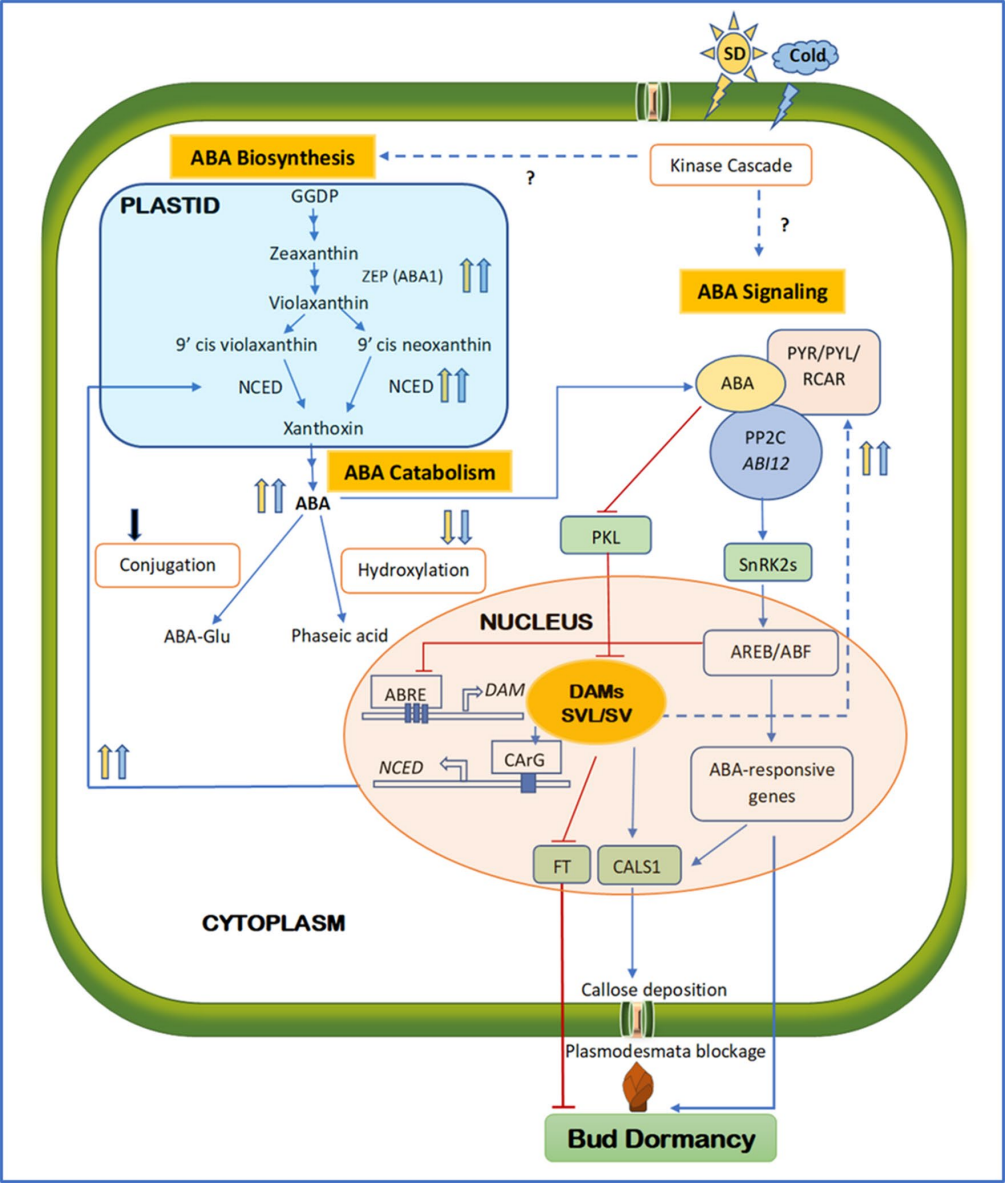
Schematic diagram integrating major components of ABA biosynthesis, signaling, and catabolism during the establishment of bud endodormancy. Solid and dashed lines indicate direct and indirect regulation; respectively. Arrowed blue and barred red lines indicate activation and inhibition, respectively. Upward and downward arrows indicate upregulation and downregulation, respectively. Blue and yellow arrows indicate cold- and SD-mediated activation, respectively, whereas the black arrow indicates an undetermined signal source. Question marks indicate the unconfirmed mechanisms. Towards the end of the growing season, SD photoperiod and short-term low temperature activate both ABA biosynthesis and signaling, possibly *via* kinase cascades. As a result, the key components in ABA biosynthesis and signaling such as ZEP, NCED, PYR/PYL/RCAR, and PP2C are upregulated, whereas ABA catabolism through hydroxylation or conjugation is downregulated. High ABA levels activate DAM and SVP/SVL transcription factors through repressing PKL. DAM promotes ABA synthesis by binding to the CArG motif of the NCED promoter, whereas SVL enhances ABA signaling by upregulating ABA receptor RCAR/PLY. The positive feedback loop between ABA and DAM/SVL can be balanced by the ABA signaling component ABRE, which represses the activity of DAM by binding to the three ABRE motifs in its promoter region. SVL can induce the expression of CALS1 and FT by binding to their promoters. The ABA signaling pathway can also upregulate CALS1, which in turn produces callose at the plasmodesmata to block the intercellular communication, contributing to the establishment of endodormancy. Activated ABA responses, repressed FT, and callose deposition all lead to the establishment of endodormancy. ABA, abscisic acid; FT, flowering locus T; SD, short day; ZEP, zeaxanthin epoxidase; NCED, 9-*cis*-epoxycarotenoid dioxygenase; PYR, regulatory pyrabactin resistance; PYL, pyrabactin like; RCAR, components of ABA receptors 1; PP2C, protein phosphate 2C; DAM, dormancy-associated MADS; SVP, short vegetative phase; SVL, SVP-like

Several recent studies have indicated that ABA may regulate dormancy through modulating the intercellular communication. In plants, the cell-to-cell transport in the symplastic continuum relies on the connectivity of specialized channels between adjacent cells called plasmodesmata. The passage of mobile molecules across the plasmodesmata is primarily controlled by the deposition and degradation of callose, a β-1,3-glucan polymer, which are catalyzed by callose synthase (CALS1) and glucanase, respectively (Wu et al., 2018b). Symplastic closure caused by callose deposition is a major mechanism in plants to defend pathogen invasion. In fact, it is also a critical step in the establishment of dormancy and is triggered by SD photoperiod events (Rinne and Schoot, 2003). This indicates that plasmodesmata constriction is a common mechanism shared by defense and dormancy and plasmodesmata is “at the crossroads” of these two events (Rinne and Schoot, 2003). In a recent study, ABA-mediated plasmodesmata constriction in hybrid aspen was shown to prevent dormancy release by limiting the passage of growth factors such as flowering locus T (FT) into the dormant buds (Tylewicz et al., 2018). In this study, an ABA-insensitive mutant (*abi1-1*) failed to produce plasmodesmatal callose and exhibited compromised dormancy in SD treatment, whereas the plasmodesmata closure and dormancy were restored through downregulation of a chromodomain remodeling factor *PICKLE* (*PKL*) or ectopic expression of *plasmodesmata located protein 1* (*PDLP1*); PKL is a chromatin remodeler that facilitates epigenetic marks (e.g. histone H3 lysine 27) and represses expression of tissue-specific genes associated with developmental transitions (Zhang et al., 2012a), and PDLPs are important regulators of plasmodesmata permeability and symplastic transport (Lim et al., 2016). It has been shown that ABA can also induce the expression of *callose synthase 1* (*CALS1*) by suppressing the expression of *PKL* (Singh et al., 2019). Taken together, these results suggest that plasmodesmata blockage is an integral mechanism in the establishment of dormancy and ABA is central to the regulation to this process (**Figure 1**).

### ABA and Cell Cycle

Cell cycle is closely related to bud dormancy. In cell cycle, G1 is the interphase at which cells accomplish most of the growth and preparation for DNA synthesis, and it is also the phase where the cells may exit from cell cycle and enter stasis of G0. Previous studies indicate that cell cycle arrests at the G1 stage in the dormant buds (Devitt and Stafstrom, 1995; Gutierrez et al., 2002). Swiatek et al. (2002) found that ABA prevents DNA replication, keeping the cells in the G1 stage, and this effect of ABA on cell cycle seemed to be limited to the transition from the G1 to S phases. Further, SD photoperiod has been shown to keep more cells in the G1 stage than the G2 stage and is associated with an increase in ABA levels (Rohde et al., 1997; Welling et al., 1997). Thus, this ABA-regulated G1/S transition appears to be a primary checkpoint for the control of ED. Previous literature also indicates that cell cycle progression is primarily controlled by cyclins (especially types A, B, and D) and cyclin-dependent kinases (CDKs), whose activity can be repressed by inhibitor of CDK/kip-related protein (ICK) and thus induces arrested or delayed cell cycle (Yamaguchi et al., 2000; Verkest et al., 2005; Lipavska et al., 2011; Torres Acosta et al., 2011) (**Figure 1**). Recently, Vergara et al. (2017) showed that ABA suppresses the expression of *CDK* (e.g. *VvCDKB1* and *VvCDKB2*) and cyclins (e.g. *VvCYCA1*, *VvCYCA2*, and *VvCYCA3*) and upregulates the expression of *ICKs* (e.g. *VvICK5*) in grapevine (*V. vinifera*) buds. Furthermore, treatments buds by the bud-breaking chemical HC reduced the content of ABA and upregulated the expression of *CCG*, and this effect was restored by the application of exogenous ABA. Together, these findings indicate that ABA-modulated cell cycle arrest may be central to the overall development of ED in woody buds.

## Gibberellins

GAs are a large group of tetracyclic diterpenoid compounds that exert significant effects on a broad spectrum of biological processes in plants. Of all the numerically coded GAs, only a few have been found to have bioactivity. These include GA1, GA3, GA4, and GA7, with GA3 being referred to as the gibberellic acid (Sun, 2008). GAs promote both vegetative and reproductive growth in plants by modulating leaf morphology, stem elongation, sex expression, seed germination, floral development, and dormancy (Takatsuka and Umeda, 2014; Hedden and Sponsel, 2015).

### GA Regulation During Dormancy

GAs are a leading phytohormone that modulates bud dormancy as significant changes of bioactive GA levels before and after dormancy have been widely noted (Cooke et al., 2012). In general, GA levels are downregulated at the induction of dormancy and upregulated during dormancy release or bud burst, and such dynamics of GA contents have been reported in many woody species such as sweet cherry (*P. avium*), hybrid aspen (*Populus tremula* × *Populus tremuloides*), grapevine, and Japanese apricot (*P. mume*) (Duan et al., 2004; Rinne et al., 2011; Zhuang et al., 2013; Zhuang et al., 2015; Zheng et al., 2018). At the early stage of dormancy initiation, decline of GA levels induces growth cessation and bud set, and growth cessation can be aborted by application of exogenous GA even with SD treatment (Mølmann et al., 2005). Interestingly, chemical inhibition of GA biosynthesis combined with low night temperature was unable to induce dormancy under long-day (LD) photoperiod in hybrid aspen (Mølmann et al., 2005), suggesting that GAs may function downstream the SD photoperiodic perception-mediated pathways for dormancy initiation. The role of GAs (especially GA3 and GA4) in promoting bud dormancy release is also evident and has been demonstrated in several woody species (Zhuang et al., 2013). In *Populus*, exogenous GA4 was found to substitute for the effect of chilling by upregulating several chilling-responsive genes (e.g. *FT*) and induce bud burst (Rinne et al., 2011). In a recent study, however, GAs’ effect in enhancing primordial growth and bud break was found to be restricted after meristem activation, and premature increase of GA rendered inhibitory effects on bud break, indicating the distinct functions of GA at the dormancy–growth transition (Zheng et al., 2018).

### GA and Dormancy Release

Studies examining exogenous GAs’ effects on dormancy release have proposed several mechanisms that GAs activate. First, GAs control dormancy cycle through modulating the intercellular communication. In plants, dormancy cycle progression is highly dependent on the mobility of molecules such as FT, auxin, sugars, and possibly ABA (Cooke et al., 2012), and thus, the permeability of plasmodesmata may play a role in controlling dormancy. A recent study indicated that SD photoperiod can trigger an ABA-mediated callose accumulation and GA catabolism (Singh et al., 2019). When active GA content is extremely low during the deep dormancy period, the rate of substance exchange in buds with adjacent organs declines remarkably (Hao et al., 2017). The connectivity of the plasmodesmata channel can be restored by GA4, which can induce the expression of β-1,3-glucanase to hydrolyze callose (Rinne et al., 2011). In a recent RNAi study, Singh et al. (2019) proposed a model that links GA to the ABA-mediated plasmodesmata closure. In this model, the authors proposed that in the absence of ABA, PKL suppresses the expression of *SVL*, a transcription factor orthologous to *Arabidopsis* floral repressor (*SVP*), which activates the expression of *CALS1* and *GA2 oxidase* (a GA catabolic gene). Upon exposure to SD photoperiod, elevated ABA level suppresses the expression of *PKL*, which in turn leads to the activation of SVL and subsequently GA catabolism and callose accumulation (Singh et al., 2019). Secondly, GA was found to enhance the production of reactive oxygen species (ROS), which are of particular importance in dormancy breaking (Zhuang et al., 2013). In grapevine (*V. vinifera*), Sudawan et al. (2016) showed that bud break rates are highly correlated to the rapid accumulation of ROS in the spring. Production of ROS at dormancy release has been documented in several plant systems and considered to play a central role in bud break (Sudawan et al., 2016; Liu et al., 2017; Beauvieux et al., 2018). Finally, GA may activate the metabolic pathways leading to dormancy release. For instance, in Japanese apricot (*P. mume*), GA4 treatment led to the enhancement of many energy metabolism pathways, including those associated with sugar metabolism (Zhuang et al., 2015). Soluble sugars are considered to be an important energy source to sustain bud growth during dormancy release. Additionally, sucrose is a potential signaling element that can indirectly enhance the expression of the genes that are related to cell division and cell cycle (Ruan et al., 2010).

### GA Metabolism During Bud Dormancy

The pathways of GA biosynthesis and catabolism have been extensively investigated by a combination of biochemical and molecular techniques. In GA biosynthesis, three classes of enzymes have been identified that correspond to the three stages of conversion. In the first stage, GA precursor geranyl geranyl diphosphate (GGDP) is converted to *ent*-kaurene *via* copalyl diphosphate synthase (CPS) and *ent*-kaurene synthase (KS) in plastids. Next, *ent*-kaurene is oxidized to GA12 through stepwise oxidation *via* two cytochrome P450 monooxygenases: *ent*-kaurene oxidase (KO) and *ent*-kaurenoic acid oxidase (KAO). In the final step, GA12 is converted to bioactive GAs including GA1, GA3, and GA4 by dioxygenases 20-oxidase (GA20ox) and 3-oxidase (GA3ox) (Sun, 2008). The GA biosynthesis occurs primarily within the vicinity of its action (Kaneko et al., 2003; Yamaguchi, 2008), so higher transcript levels of the majority of GA biosynthetic genes can be found in the actively growing tissues including the bursting buds. Unlike the GA biosynthetic pathway that involves various enzymes and multiple stages of conversion, GA catabolism occurs mainly through 2-β hydroxylation *via* enzyme GA 2-oxidase (GA2ox) (Fleet and Sun, 2005). Two novel mechanisms that were found to reduce the bioactivity of GAs are the epoxidation by a P450 monooxygenase (Zhu et al., 2006) and methylation (Varbanova et al., 2007).

The metabolic enzymes of GA are crucial players in the maintenance of GA homeostasis and the regulation bud dormancy. Specifically, the synthetic genes *GA20ox* and *GA3ox* and the catabolism gene *GA2ox* are of particular importance in regulating GA levels, and they are all encoded by multigene families. Transcription studies have shown the close correlation between the expression of *GA20ox*, *GA3ox*, and *GA2ox* and the GA level variation during bud dormancy in woody species such as rose (*Rosa* sp.) (Choubane et al., 2012), Japanese apricot (*P. mume*) (Wen et al., 2016), and grapevine (Zheng et al., 2018). In particular, the expression of *GA2ox* was found to be significantly upregulated by SD photoperiod, and its overexpression resulted in accelerated bud set and delayed bud flush (Zawaski et al., 2011). This indicates that the *GA2ox*-mediated catabolism is the key mechanism that reduces GA levels for the establishment and maintenance of bud dormancy. In a comprehensive transcription study in the tea plant *Camellia sinensis*, the expression of the GA synthetic enzyme genes *KAO* and *KO* was in line with the progression of bud dormancy (Yue et al., 2018). When multiple genes from the *GA20oxs*, *GA3oxs*, and *GA2oxs* families were examined, some genes were expressed in correlation with the bud dormancy progression, and others showed differential expression (Yue et al., 2018). Similarly, these genes were differentially expressed when tea plants were treated with exogenous GA3. Such redundant roles and possibly specialized functions of GA metabolism gene families contribute to the complexity of the underlying mechanism of GA metabolism.

### GA Signaling in Bud Dormancy

In the GA signaling pathway, the GA-GID1-DELLA module is considered to be universal and highly conserved in angiosperms (Sun, 2011). In this model, DELLA proteins belong to the GRAS transcriptional factor family and negatively regulate GA responses in the absence of GA. GID1 (GA-insensitive dwarf 1) is the GA receptor and can form the GA-GID1 complex *via* binding with a GA-specific F-box protein SLEEPY1 (SLY1) to trigger rapid degradation of DELLA proteins *via* the ubiquitin-proteasome pathway (Itoh et al., 2003; Ariizumi et al., 2013; Hauvermale et al., 2014). Recently, DELLAs were also found to be involved in the GA feedback mechanism by upregulating the GA biosynthesis gene *GA20ox* (Yoshida and Ueguchi-Tanaka, 2014). In addition to GA metabolism, recent genetic studies also indicated the involvement of the GA signaling pathway in dormancy. For example, the expression of *GID1* in Chinese cherry (*Prunus pseudocerasus*) is significantly downregulated at the early stage of dormancy development and rapidly increases when buds enter the ECD stage (Zhu et al., 2015). Similar increase of *GID1* expression prior to bud break was also found in tea plant (Yue et al., 2018). The parallel relationship between *GID1* expression and GA levels confirms the role of GID1 as a positive mediator of GA responses. In contrast to GID1, DELLA proteins mainly act as a negative mediator of GA responses. In poplar (*P. tremula* × *Populus alba*), *DELLA* genes (*GAI* and *RGL*) were found to be upregulated in response to SD photoperiod (Zawaski and Busov, 2014). Similarly, high *DELLA* expression in tea plant was observed during the induction of dormancy (Yue et al., 2018), and overexpression of *C-repeat binding factors* (*CBFs*), a regulatory gene responsible for cold acclimation, led to upregulation of DELLA and reduced growth in apple (Wisniewski et al., 2015). Thus far, it is still unclear if these GA signaling genes respond directly to the environmental signals or if their expression merely reflects the developmental events during dormancy.

### GA–ABA Cross Talks During Dormancy

The antagonism between ABA and GA marks the main feature of their interactions in modulating biological processes, in which the metabolism and signaling of these two phytohormones respond oppositely to environmental cues. In dormancy, the levels of GA and ABA are inversely correlated, with the ABA/GA ratio varying in parallel with the depth of dormancy (Duan et al., 2004). For example, in Japanese apricot, the decrease of ABA level is accompanied by a gradual increase in GA level from dormancy through dormancy release (Wen et al., 2016). Transgenic studies showed that the alternation of one hormone may affect the metabolism of the other. Mutations in the *ABA* pathway lead to higher GA content by promoting the GA synthesis genes *GA3ox*, and inhibition of GA upregulates the ABA synthetic genes (*ABA1* and *NCEDs*) while downregulating the ABA catabolic gene *CYP707A2* (Seo et al., 2006; Oh et al., 2007). These results suggest that ABA and GA are involved in the metabolic regulation of each other. Further evidence was obtained in a recent transcriptome study with tea plant (Yue et al., 2018), in which GA treatment was found to repress the expression of both ABA biosynthetic genes (*ZEP*, *NCED1*, and *NCED1*) and catabolic gene (*CYP707A2*). In the same study, ABA treatment was found to upregulate the GA synthetic genes (*CsKS*, *CsKAO*, and *CsKO* and *GA3ox1*, *GA20ox1*, and *GA20ox2*), while the catabolic gene *GA2oxs* showed different expression patterns upon exposure to ABA in a concentration-dependent manner.

**Figure 2:**
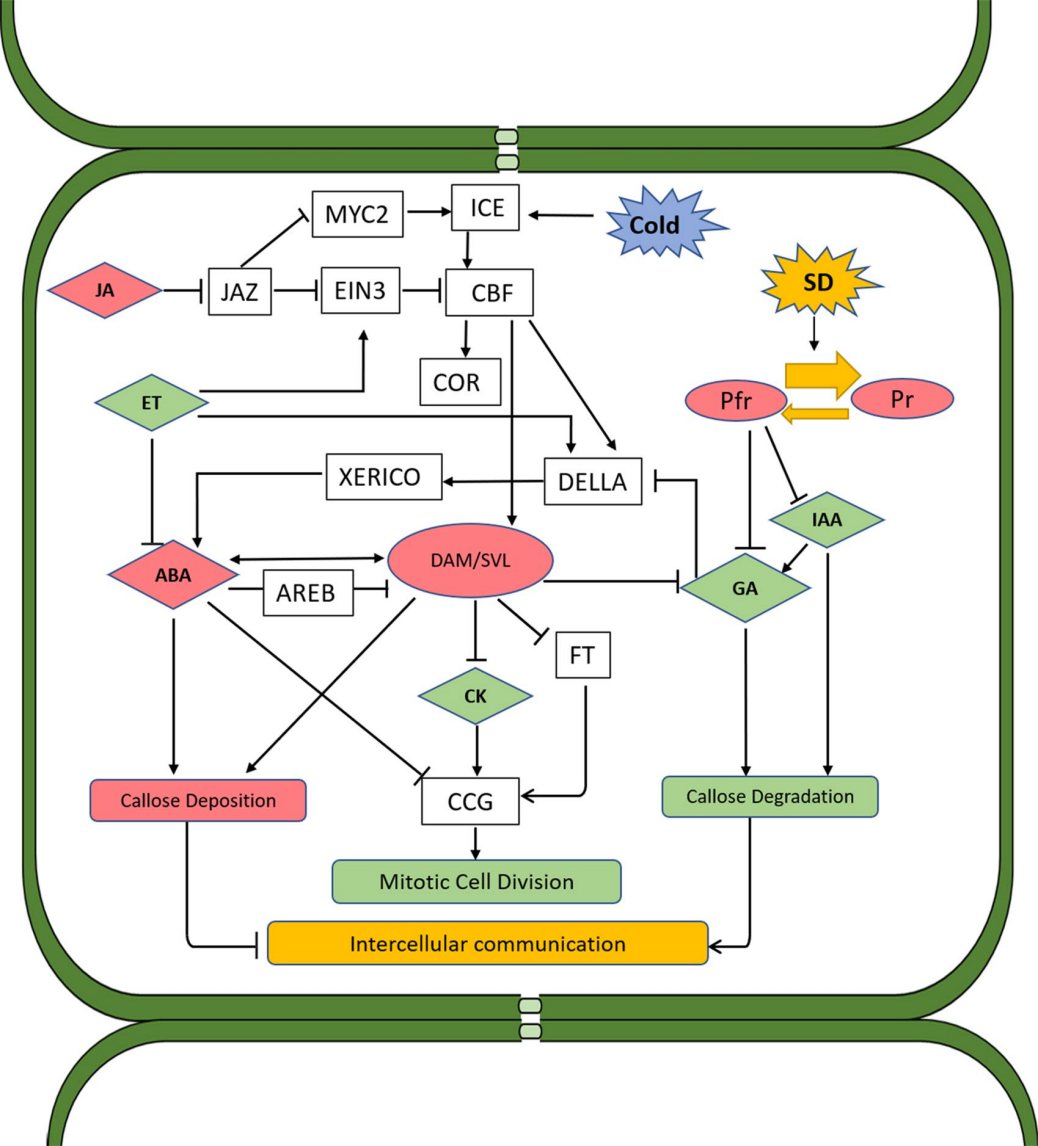
Proposed schematic model of hormone interactions during bud dormancy induction and release in woody perennials. Solid arrows and lines indicate actions or interactions among hormones, pathways, and environmental cues that have been documented in the literature. Red color indicates the substance or process that induces dormancy, and green color indicates those that promote dormancy release. Both SD photoperiod and low temperature induce CBF, which directly promotes the expression of *DAM* genes. DAM proteins regulate dormancy through ABA-dependent and ABA-independent pathways. In the former pathway, accumulation of DAMs reduces GA level, thus activating DELLA and subsequently XERICO proteins, which in turn promotes ABA synthesis. *DAM* genes can also upregulate ABA levels through upregulating the gene encoding NCED, a key enzyme in ABA synthesis. In addition to the control of DAMs over ABA biosynthesis and signaling, ABA can also negatively regulate the expression of DAM genes through SnRK2 and the ABA signaling component AREB. This pathway probably serves as a negative feedback regulation mechanism. In the ABA-independent regulation pathway, DAM proteins induce dormancy through negative regulation of FT, which in turn prevents CCG-mediated dormancy release. ABA can also repress CCG and inhibit the mitotic cell division. ABA was also found to suppress the intercellular communication during dormancy by enhancing the expression of callose synthase, leading to callose deposition and blockage of plasmodesmata. This alteration of plasmodesmata during dormancy is reversed by GA as buds transition to bud break. GAs induce the expression of glucanases which degrade callose, allowing for the passage of sugars and other growth-promoting factors. Under SD conditions, GA biosynthesis is inhibited through phytochrome and phytochrome-interacting factors. ET is induced by SD and has a negative impact on GA biosynthesis and signaling, which nominates it as a dormancy inducer. However, ET also inhibits ABA synthesis and signaling and negatively regulates CBF through activation of EIN3, which is also subject to the regulation of JA through JAZ proteins. JA induces bud dormancy by targeting JAZ proteins for degradation *via* the ubiquitination/26S proteasome, which in turn releases MYC2 and ICE1 from repression. Both MYC2 and ICE1 activate the expression of CBF. CK can repress ABA and promote dormancy release through inducing the expression of *CCG*s. IAA facilitates dormancy release through promoting GA biosynthesis and callose degradation. ABA, abscisic acid; CBF, C-repeat binding factor; CCG, cell cycle gene; CK, cytokinin; ET, ethylene; EIN3, ETHYLENE INSENSITIVE 3; FT, flowering locus T; GA, gibberellins; JA, jasmonates; IAA, indole-3-acetic acid; SD, short day; NCED, 9-*cis*-epoxycarotenoid dioxygenase; DAM, dormancy-associated MADS.

The interaction between ABA and GA also occurs at the signal transduction level. In addition to playing a central role in the GA signaling pathway, DELLA proteins also act as a cross talk node that integrates the signaling pathways of several other hormones including ABA (Santner and Estelle, 2009; Davière and Achard, 2016). In mutants with impaired GA signaling, accumulated DELLAs upregulate the expression of *XERICO*, a RING zinc finger protein known to induce ABA synthesis (Zentella et al., 2007; Ariizumi et al., 2013) (**Figure 2**). In tea plant, GA treatment induces the expression of a negative mediator *PP2C* in ABA signaling, while represses the ABA receptor *PYL8* (Yue et al., 2018). The fact that DELLA proteins can be stabilized by stress-induced ABA (Ariizumi et al., 2013) indicates that GA responses may be inhibited by high ABA levels at the dormancy initiation. Recently, Yue et al. (2018) showed that exogenous ABA represses the expression of GA receptor *GID1* and induces the expression of *DELLA* genes. However, long hours of ABA treatment were found to suppress *DELLA* expression.

## Ethylene

Although it is recognized as the ripening hormone, ET has wide-ranging effects on a number of other biological processes including, but are not limited to, seed germination, flowering, abscission, senescence, and stress responses (Bleecker and Kende, 2000). The function of ET in dormancy is closely related to its biosynthesis and signaling transduction. ET biosynthesis starts with the conversion of methionine to *S*-adenoysl-methionine (SAM) by SAM synthetase (Iqbal et al., 2013). In the subsequent rate-limiting and committed step, SAM is converted to 1-aminocyclopropane-1-carboxylic acid (ACC) by ACC synthase (ACS). In the final step, oxidization of ACC by ACC oxidase (ACO) produces ET, hydrogen cyanide (HCN) and CO_2_ (Wang et al., 2002a). In this biosynthetic pathway, enzymes ACS and ACO are the important regulators of endogenous content of ET and are both encoded by multi-gene families (Van de Poel and Van Der Straeten, 2014); (Johnson and Ecker, 1998). During high rates of ET biosynthesis, accumulated cyanide can become toxic to plants, and this toxicity can be alleviated by converting cyanide to β-cyanoalanine by enzyme β-cyanoalanine synthase (β-CAS) (Wang et al., 2002a). In the ET signaling pathway, five ET receptors ETR1, ETR2, EIN4, ERS1, and ERS2 have been identified in *Arabidopsis* (Wang et al., 2002a). In the absence of ET, these receptors recruit Constitutive Triple Response1 (CTR1) and inhibit a membrane spanning protein EIN2, a positive regulator of ET signaling (Gao et al., 2003; Qiu et al., 2012). EIN2 stabilizes the downstream transcription factors ETHYLENE INSENSITIVE 3 (EIN3)/EIN3-Line1 (EIL1) by inducing proteasomal degradation of F-box proteins (EBF1 and EBF2), which mediate proteolysis of EIN3/EIL1 in the absence of ET (An et al., 2010). Among the EIN3 direct targets are the *ET RESPONSE FACTOR* (*ERF*) genes, which belong to the AP2/ERF superfamily, members of which play pivotal roles in adaptation to various biotic and abiotic stresses (Mizoi et al., 2012).

The results of several studies have converged to indicate ET and its response pathway is involved in dormancy regulation. Early evidence showed that ET levels are elevated at both dormancy initiation and bud break stages and application of ET antagonist 2,5-norbornadiene (NBD) causes premature dormancy break in potato microtuber (Suttle, 2004). Later on, Ruonala et al. (2006) reported that ET-insensitive mutation (*etr1-1*) causes abolished formation of terminal buds and biosynthesis of ABA and delayed dormancy in European white birch (*Betula pendula*) in SD photoperiod condition. Similar result was also found in chrysanthemum, in which mutants with impaired ET receptor gene (*DG-ERS1*) fail to enter dormancy at dormancy inducing temperature (Sumitomo et al., 2008). The requirement of ET in the induction of dormancy was further confirmed by microarray and transcriptomic studies, in which ET biosynthesis gene set and signaling component genes (e.g. *ETR2*, *EIN3 EIN4* and *ERF*) in poplar are upregulated in dormancy inducing conditions (Ruttink et al., 2007; Howe et al., 2015). Investigations into the interaction between ET and GA indicated that ET may modulate GA or GA signaling pathway to induce dormancy. It has been noted that in responses to environmental stresses, activated ET signaling induces GA deactivation and accumulation of DELLAs through enhancing the expression of ET Response Factors (*ERF6*) (Dubois et al., 2013; Dubois et al., 2015). Moreover, Achard et al. (2007) reported that ET can act on and stabilize DELLA *via* CTR1, independent of GA mediation. More evidence of the interaction between ET and GA was obtained when the interaction between ET action and phytochrome signaling was examined. In tobacco, low red to far-red light ratios (R:FR) was found to trigger ET biosynthesis and ET insensitive transgenic lines exhibit no shade avoidance, which can be rescued by application of GA3 (Pierik et al., 2004). This suggests that the early signal transduction of phytochrome-mediated light responses might trigger ET accumulation and GA reduction in response to SD, leading to growth cessation and dormancy inductions.

Interestingly, ET was also indicated to participate in the dormancy release. Application of ET signaling inhibitor NBD leads to increase of ABA levels in breaking buds, suggesting ET is required for the degradation of ABA and modulating of ABA signaling (Ophir et al., 2009; Zheng et al., 2015). In *Arabidopsis*, ET negatively regulates the cold tolerance gene *CBF*, through activating EIN3, which in turn represses *CBF* genes by binding to their promoters (Shi et al., 2012) (**Figure 2**). Further evidence was supported by the following observations in grape buds: 1) ET biosynthesis can be temporarily activated by dormancy break stimuli such as HC, heat shock and sodium azid; 2) exogenous ET application enhances bud break; and 3) dormancy release is severely delayed when of ET signaling is blocked by NBD (Ophir et al., 2009). These findings suggest that ET has complex actions during bud dormancy, in which ET interacts with ABA synergistically during dormancy initiation, but antagonistically during dormancy release. These studies also suggest that ET and its signaling pathway is essential for the development of dormancy, and both ABA and GA respond downstream of ET mediated dormancy regulation. How these opposite processes are integrated in a stage-dependent way warrants further investigation.

Increasing evidence has suggested that bud break is triggered by elevated levels of ROS, and ET is actively involved in this process. Differential expression of genes induced by HC revealed connection between dormancy release and oxidative stress, hypoxia, mitochondrial activity, ET biosynthesis and signaling pathways (Ophir et al., 2009; Sudawan et al., 2016; Ionescu et al., 2017). In response to HC, plants transiently elicit ROS, such as H_2_O_2_, and subsequently activating many pathways that are related to dormancy release, including antioxidant systems. ET biosynthesis has been suggested to increase oxidative stress in plants due to production of hydrogen cyanide (Ionescu et al., 2017), and hydrogen cyanide was found to be more effective than ET in breaking buds (Ionescu et al., 2017). On the other hand, ET can also act as a messenger molecule to activate the antioxidant system (such as CAT) to scavenge excessive ROS (Vergara et al., 2017). Indeed, the accumulation of ROS and their elimination has been proposed to be pivotal steps in releasing dormancy (Sudawan et al., 2016; Beauvieux et al., 2018).

## Cytokinins

CKs are a group of adenine-derived small compounds that play important roles in a variety of plant processes including cell division, cell differentiation, apical dominance, leaf senescence, and stress tolerance (Sakakibara, 2006; Zürcher and Müller, 2016). The effects of CKs are highly dependent on cell and tissue types, developmental stage and environmental conditions, thus CKs are particularly important in modulating meristem activity and morphogenesis. At the cellular level, CKs can activate cell cycle regulator, CDK by dephosphorylating its tyrosine (Tyr), and this CK effect is considered to be primary and required for the proper progression of cell cycle, which would otherwise arrest at the G2 phase (Zhang et al., 2005). Natural CKs differ greatly in the side chains, which are attached to the parental compound adenine, and this structural diversity provides high specificity of the interaction between CKs and the receptors (Kieber and Schaller, 2014). In CKs metabolism, the rate-limiting step in biosynthesis pathway is mediated by the adenosine phosphate-isopentenyltrasferase (*IPT*), and the major catabolic step is the oxidation of CKs that removes the side chains by the CK oxidase/dehydrogenase1 (*CKX*). Though CKs are highly mobile in plants and can be transported in the xylem sap over long distances, locally synthesized CKs were suggested to be critical in regulating dormancy (Tanaka et al., 2006).

Similar to its effect in releasing latent buds from PD, CKs are also implicated in the regulation of dormancy release (Faust, 1997). Early research showed that CK concentration in xylem sap increases rapidly in response to bud breaking chemicals and reaches a maximum level at the budburst in apple (Cutting et al., 1991). Genetic analysis demonstrated that forced condition either through LD photoperiod or treatment with HC can increase CK levels in the grapevine cuttings by activating the expression of CK biosynthesis genes (*IPT* and lonely Guy *LOG1*) while downregulating that of CK catabolism gene *CKX* (Noriega and Pérez, 2017). Furthermore, temporal expression analysis showed that the increase of CK levels induced by forced conditions can upregulate the expression of cell cycle genes and subsequently raises the rate of cell division and cellular respiration, which are the major events preceding the activation of dormant buds (Noriega and Pérez, 2017). In support of these findings, Hartmann et al. (2011) showed that high levels of CK in *IPT-e*xpressing potato tubers promoted early dormancy release, whereas reduced CK contents through *CKX* overexpression inhibited cellular metabolism and cell proliferation, rendering no response to GA3 and leading to prolonged dormancy period. Another evidence of CK’s effect in dormancy release lies in its mediation of light signal. Previous studies have identified several His kinases as CK receptors such as AHK2, AHK3 and AHK4, which positively regulate CK signals by phosphorylating His-containing phosphotransfer proteins (Higuchi et al., 2004; Mahonen et al., 2006; Riefler et al., 2006). Using loss-of-function approach, Tran et al. (2010) showed that these CK receptors negatively regulate the ABA responses in the presence of CK. Dobisova et al. (2017) demonstrated that light can regulate the transcription of these CK receptors to control the CK signaling. This result was supported by the finding in rose (*Rosa hybrida*), in which CK was found to participate in the initial responses of the light signaling pathway that promotes bud outgrowth (Roman et al., 2016). These results suggest the light-mediated increase of CKs during the dormancy release may contribute to reduce ABA levels. Taken together, these results suggest that CK is an essential regulator in the dormancy release and CK acts upstream of GA and ABA response pathways in stimulating meristematic activity.

## Auxin

Auxin has long been known to promote stem elongation and to suppress the growth of lateral buds, in a phenomenon of the apical dominance. Recent findings indicate auxin is also involved in plant senescence, blooming and stress responses (Di et al., 2016; Fendrych et al., 2016). Of all the four types of naturally occurring auxins, IAA is the most abundant and relatively well studied (Simon and Petrášek, 2011). IAA biosynthesis involves a two-step conversion: in the first step, IAA precursor tryptophan is converted to intole-3-pyrunvae (IPA) by an amino transferase; in the second step, IPA is oxidized to IAA by flavin monooxygenase (YUC) and this reaction is a rate-limiting step in IAA synthesis pathway (Zhao, 2012). Auxin biosynthesis occurs primarily in shoot apex and young leaves. Auxin is transported basipetally (from tip to base) through specialized membrane carriers, called PIN-FORMED proteins (PIN), which also play a role in maintaining auxin homeostasis (Friml and Palme, 2002; Muller and Leyser, 2011). In the auxin signaling transduction pathway, Aux/IAAs are transcriptional repressors that bind to and inhibit the activity of *AUXIN RESPONSE FACTORs* (*ARFs*), in the absence of auxin. ARF can bind to the Auxin Response Elements (AREs) in the promoter region of the auxin responsive genes, and both activaor AFRs and repressor ARFs have been identified (Leyser, 2018). In the presence of auxin, Aux/IAAs are subjected to the auxin triggered Ubiquitin degradation.

Being a major growth promoter, IAA has been implicated in the dormancy release in many species. Early study showed that exogenous auxin can promote the degradation of dormancy callose in the phloem of magnolia (*Magnolia kobus*) and lead to the restoration of the symplastic paths (Aloni and Peterson, 1997), which is the preparatory step of bud break. In tea plant (*C. sinensis*), Nagar and Sood (2006) found that IAA levels remain low during the entire stage of dormancy and increase steadily after dormancy release until bud break in the spring. This study also revealed that free IAA content changes in opposite to its conjugated form, suggesting conjugation may serve as a major mechanism in maintaining the homeostasis of endogenous IAA during dormancy. Increases of IAA levels during dormancy release were also reported in Chinese fir (*Cunninghamia lanceolata*) (Qiu et al., 2013) and Chinese plum (*P. mume*) (Zhang et al., 2018). Considering the fact that polarly transported IAA induces GA biosynthesis required for growth (Wolbang and Ross, 2001; Wolbang et al., 2004; Frigerio et al., 2006), IAA may likely facilitate dormancy release in collaboration with GA. Genetic study suggests that the IAA levels is mainly controlled at the metabolic level, as the of IAA synthetic gene *YUC3* is upregulated during natural dormancy release or by HC; high levels of IAA, along with CK switches on the cell cycle machinery and release the buds from dormancy (Noriega and Pérez, 2017). Transcriptomic data revealed differential expression of the main components in the IAA signaling pathway during the transition from dormancy to active growth, and their potential roles remain yet to be further elucidated (Qiu et al., 2013; Zhang et al., 2018).

On the other hand, IAA also appears to be an integrator of environmental signals during the dormancy establishment. When exposed to low temperature or in combination with SD photoperiod, IAA levels in strawberry and the transcript levels of polar auxin transport (PAT)-related gene (e.g. *PIN*) decline significantly, accompanied by an increase of ABA levels and global genomic DNA methylation, indictive of dormancy initiation (Zhang et al., 2012b). In exposure to SD photoperiod, the auxin signaling repressor AUX/IAAs and PAT-related genes of hybrid aspen lose their responsiveness to auxin, accompanied by the downregulation of activator AFRs and induction of repressor ARFs (Baba et al., 2011). Transcriptome data implied that the majority of auxin-associated genes are downregulated during dormancy in Japanese apricot and poplar (Zhong et al., 2013; Howe et al., 2015). These results suggest that modulation of cellular auxin content, auxin responsiveness, auxin transport capacity, and conjugation could all be integrated in the regulation network of dormancy.

## Jasmonates

JAs are a class of lipid-based plant hormones that regulate diverse processes in plants development and defense (Browse, 2009). Bioactive forms of JA include jasmonic acids, its biosynthetic precursor 12-oxophytodienoic acid (OPDA) and the conjugate form JA-Ile, which all have been shown to be effective signaling compounds. Similar to ABA, JAs prevent plant growth by repressing meristem activity, and some stress related genes can be activated by both JAs and ABA, indicating their synergism in certain processes (Swiatek et al., 2002; Delker et al., 2006; Zhang and Turner, 2008; Browse, 2009). Jasmonic acids can arrest cell cycle of tobacco (*Nicotiana tabacum*) BY-2 cells at the G1 or G2 phases by repressing the activity of CDK (Swiatek et al., 2002). This may explain the reason that jasmonic acid and methyl JA (MeJA) inhibit seed germination in many species (Linkies and Leubner-Metzger, 2012). Indeed, JA can induce leaf senescence and control the expression of senescence-related genes in many species (Ueda and Kato, 1980; Shan et al., 2011; Yan et al., 2012; Lee et al., 2015). In *Arabidopsis*, JA biosynthesis is subject to the control of *TEOSINTE BRANCHED/CYCLOIDEA/PCF* (*TCP*) transcription factors, which can directly activate JA biosynthetic gene *LIPOXYGENASE2*, and when *TCP4* is targeted by microRNA (*miR319*), JA synthesis is repressed and leaf senescence is delayed and the expression of senescence associated genes decreased (Schommer et al., 2008). However, JAs seem to have opposite effects during bud dormancy. In beech trees (*Fagus sylvatica*), JA levels were found to increase remarkably during bud burst (Juvany et al., 2015). In agreement with this, contents of JA-Ile in potato tubers increase gradually as the buds transition from dormancy to active sprouting (Suttle et al., 2011; Juvany et al., 2015). Transcriptomic analysis showed that JA pathway is repressed during dormancy but activated during ECD stage and bud break (Hao et al., 2017). These results suggest that JAs may play a role other than inhibiting growth during dormancy release.

Recent research indicates that JA signaling pathway is actively involved in cold acclimation process, which is closely related to dormancy. In JA signaling pathway, JA ZIM-DOMAIN (JAZ) proteins are key repressors of JA signaling, and can form a JA coreceptor complex with F-box protein COI1 (CORONATINE INSENSITIVE1) (Sherif et al., 2015; Hu et al., 2017). Upon binding of JA to the complex, JAZ proteins undergo ubiquitin-mediated degradation and release several groups of transcription factors, such as the helix–loop–helix (bHLH) factors MYC2, MYC3 and MYC4 (Goossens et al., 2016). It has been shown that MYC2, as a key transcriptional activator of JA responses, can activate gene *SAG29* (*SENESCENCE-ASSOCIATED GENE29*) from *Arabidopsis* by binding to its promotor and promote JA-induced leaf senescence, whereas another group of bHLH transcription factors (bHLH03, 13, 14 and 17) can bind to the promotor of *SAG29* to counteract the enhancing effect of MYC2/MYC3/MYC4, thus these signaling components form an elegant feedback regulation mechanism to modulate JA-induced senescence (Qi et al., 2015). The pathway that is composed of the Inducer of CBF Expression (ICE), CBF transcription factors, and various Cold Regulated (COR) genes is the best-studied cold response pathway (Wang et al., 2017; Jin et al., 2018). Recently, more evidence was obtained for the direct interaction between JA signaling and the ICE-CBF-COR signaling cascade. First, the physical interactions between JA signaling repressors (JAZ proteins) and ICE proteins was demonstrated by yeast two-hybrid assays, and JA induced degradation of JAZ proteins led to the upregulation of the *COR* genes (Hu et al., 2013). Second, the JA-induced transcriptional activator, MYC2, was found to interact with ICE1 and activate the cold response pathway, reinforcing the notion that JA acts as an essential upstream signal of the ICE-CBF-COR pathway to positively regulate cold tolerance Zhao et al. (2013). Third, In *Arabidopsis*, it was noticed that exogenous JA application enhanced plant freezing tolerance and JA biosynthesis was triggered upon cold exposure (Hu et al., 2013). However, whether such cross talks between JA and cold-responsive pathways would function during dormancy, especially in woody species, still needs further validation. The interactions between JAZ proteins and other hormonal signaling components (e.g. DELLA and EIN3/EIL1) (Melcher et al., 2010; Zhu et al., 2011) indicate that JA could be widely and closely related to the regulation network of cold acclimation and dormancy, and its function will be better understood when examined in the context of its interaction with other hormones.

## *DAM* and *SVL* Genes

The Dormancy-Associated MADS-box (*DAM*) has become increasingly prominent in recent studies on the bud dormancy. The first *DAM* genes were identified in a peach mutant *Evergrowing* (*EVG*), in which deletion of *EVG* locus rendered complete loss of growth cessation and bud formation in dormancy inductive conditions (Bielenberg et al., 2004). Later, six tandemly arranged *MADS-box* genes in the *EVG* locus of peach were identified, and they were believed to be the candidates that regulate dormancy, thus named Dormancy-Associated MADS-box (*DAM*) genes (Bielenberg et al., 2008). Phylogenetic analysis indicates these *DAM* genes are homologous to two transcription factor genes of *Arabidopsis*: *SHORT VEGETATIVE PHASE* (*SVP*) and *AGAMOUS-LIKE 24* (*AGL24*) (Jimenez et al., 2009). Genes of *SVP* and *AGL24* have antagonistic functions, in which *SVP* prevents flowing through inhibition of floral promoting gene *FT*, whereas *AGL*24 activates flowering by enhancing *LEAFY* (*LFY*) gene, a major regulator of floral development of land plants (Hartmann et al., 2000; Michaels et al., 2003; Lee et al., 2008). Up to date, numerous *DAM-*like or *SVP*-like (*SVL*) genes that potentially mediate in the control of dormancy have been identified in diverse species (**Table 1**). Based on the phylogenetic data, no consensus has been reached weather *SVL* genes represent a distinct group apart from *DAMs* or they belong to one group. Nevertheless, the fact that *DAM* and *SVL* both exhibit growth inhibitory effect across different species demonstrated by transgenic studies (Sasaki et al., 2011; Wu et al., 2017a; Wu et al., 2017b; Singh et al., 2018; Singh et al., 2019; Yamane et al., 2019) suggests that their potential roles may be similar or overlapping in mediating dormancy. Specifically, functional commonality in *DAM* and *SVL* was manifested in that they both promote ABA biosynthesis by upregulating *NCED* (Tuan et al., 2017; Singh et al., 2018) and delay bud break (Wu et al., 2017a; Singh et al., 2018; Yamane et al., 2019).

**Table 1:** The chronology of select major research findings related to identification of function of *DAM/SVL* genes.

Time	Main findings	Species	References
1994	Nondormant evergreen peach identified from southern Mexico. This genotype was found to be controlled by a recessive allele (*evg*).	Peach (*Prunus persica*)	(Rodriguez et al., 1994)
2002	Genetic and physical mapping of *evg* genes.	Peach (*P. persica*)	(Wang et al., 2002b)
2004	The first *DAM* genes were recognized in the *Evergrowing* (*EVG*) mutant.	Peach (*P. persica*)	(Bielenberg et al., 2004)
2007	*SVP*-like *MADS* gene was found to be downregulated during dormancy release.	Raspberry (*Rubus idaeus*)	(Mazzitelli et al., 2007)
2008	Six tandemly arranged *DAM* genes were identified in the *EVG* locus.	Peach (*P. persica*)	(Bielenberg et al., 2008)
2008	*DAM1* and *DAM2* were found to be associated with endodormancy.	Leafy spurge (*Euphorbia esula*)	(Horvath et al., 2008)
2009	*DAM1*, *DAM2*, and *DAM4* were found to be associated with growth cessation and bud set.	Peach (*P. persica*)	(Jimenez et al., 2009)
2010	Chromatin modification in *DAM1* promoter region marked by decrease of H3K4me3 and increase of H3K27me3 after cold exposure.	Leafy spurge (*E. esula*)	(Horvath et al., 2010)
2010	Cloning and characterization of *PpMADS13-1* and *PpMADS13-2*. No linkage was found between DNA methylation and dormancy progression.	Pear (*Pyrus pyrifolia*)	(Ubi et al., 2010)
2011	Overexpression of *PmDAM6* led to growth cessation and terminal bud set.	Japanese apricot (*Prunus mume*)	(Sasaki et al., 2011)
2012	*DAM6* expression was closely correlated with the dormancy release and accompanied by a decrease of H3K4me3.	Peach (*P. persica*)	(Leida et al., 2012)
2015	Four *DAM*-like genes were characterized in apple, with two (*MdDAMa* and *MdDAMc*) coinciding with the dormancy.	Apple (*Malus domestica*)	(Mimida et al., 2015)
2015	Ectopic expression of cold response signal factor *PpCBF* was found to alter *DAM* expression.	Apple (*M. domestica*)	(Wisniewski et al., 2015)
2015	The stable gene-silencing mark H3K27me3 was found to be more enriched in *DAM1*, *DAM4*, *DAM5*, and *DAM6* gene regions in nondormant buds than in dormant buds.	Peach (*P. persica*)	(De La Fuente et al., 2015)
2016	*PpCBF* was found to enhance *PpDAM1* and *PpDAM3* transcription during dormancy induction; DAM proteins inhibit *PpFT2* transcription during dormancy release.	Pear (*P. pyrifolia*)	(Niu et al., 2016)
2017	First evidence of interaction between *DAM* and plant hormone: *PpDAM1* activates ABA biosynthesis, and ABA signaling component *ABRE* negatively regulates *PpDAM1* activity.	Pear (*P. pyrifolia*)	(Tuan et al., 2017)
2017	Overexpression of *SVL* (*SVP2*) in kiwifruit delays bud break before the completion of chilling requirement.	Kiwifruit	(Wu et al., 2017b)
2018	Cold response signal factor *PmCBF5* was found to negatively control *PmDAM* during the onset of dormancy.	Japanese apricot (*P. mume*)	(Zhao et al., 2018)
2018	*SVL* mediates bud transition into and out of dormancy through regulating essential dormancy hormones, e.g., ABA and GA.	Hybrid aspen (*Populus* spp.)	(Singh et al., 2018)
2019	*SVL* acts downstream ABA by suppressing GA and inducing callose deposition in dormancy induction.	Hybrid aspen (*Populus* spp.)	(Singh et al., 2019)

ABA, abscisic acid; GA, gibberellin; DAM, dormancy-associated MADS; SVP, short vegetative phase; SVL, SVP-like.

### *DAM/SVL* Genes Positively Regulate Dormancy

Though *DAM* genes are highly conserved and closely arranged, their expression patterns are generally distinct in response to seasonal changes and developmental signals. In peach (*P. persica*), *DAM1*, *DAM2* and *DAM4* are upregulated during growth cessation and bud set, whereas *DAM5* and *DAM6* expression is strongly induced by SD photoperiod and their transcripts increase only during dormancy, then decline upon natural or chemical induction of dormancy release (Li et al., 2009; Jimenez et al., 2010; Yamane et al., 2011). It is thus suggested that *DAM5* and *DAM6* in peach operate downstream of the circadian perception of photoperiodic stimuli, which occurs prior to the onset of chilling. As the dormancy progresses, the expression of these genes decreases concomitant with chilling accumulation and reaches the minimum at bud break. In light of these findings, *DAM5* and *DAM6* are believed to be the primary internal regulators of dormancy induction and maintenance in peach and probably other stone fruit species.

Following the identification of peach *DAM* genes, identification and characterization of more *DAM* and *SVP* homologs have been reported in other perennial species including but are not limited to raspberry (*Rubus idaeus*) (Mazzitelli et al., 2007), Japanese pear (Ubi et al., 2010), Japanese apricot (*P. mume*) (Sasaki et al., 2011), kiwifruit (*Actinidia* spp.) (Wu et al., 2012), and pear (*P. pyrifolia*) (Niu et al., 2016). In a recent review, Falavigna et al. (2018) divided the documented *DAM* and *SVP* like genes into three groups according to their expression patterns. The majority of these *DAM* genes fall in the group that are highly expressed during the intensified stage of dormancy, characteristic of peach *DAM 5* and *DAM 6*; whereas other *DAM* genes are asynchronous with the progression of dormancy. More definitive role of *DAM* genes in bud dormancy regulation were demonstrated in a number of recent transgenic studies. In poplar, Sasaki et al. (2011) showed that constitutive overexpression of Japanese apricot (*P. mume*) *DAM6* gene induced premature growth arrest and bud set. Similarly, ectopic expression of *DAM*-like gene *MdDAMb* or *MdSVP* in apple causes delayed bud break (Wu et al., 2017a). Overexpression of *DAM6* in Japanese apricot resulted in inhibited shoot growth, early bud set, repressed bud break competency and delayed bud break (Yamane et al., 2019). The high similarity of the *DAM* genes characteristics across species indicate that bud dormancy may be regulated by a highly conserved mechanism that is shared by most perennial species.

### FT and CBF in *DAM* and *SVL* Regulation

Though the role of *DAM* genes in dormancy regulation has been extensively verified, the underlying molecular processes are still largely unknown. Recent studies suggested that flowering regulating gene *FT* and cold response transcription factors CBFs may act as major mediators in the *DAM* regulation pathway. *FT* encodes a small globular protein that has been implicated in flowering, prevention of growth cessation and proper induction of dormancy (Bohlenius et al., 2006; Wigge, 2011). High expression of *DAM* genes was found to correlate with the downregulation of *FT* in leafy spurge (*Euphorbia esula*) and pear (*P. pyrifolia*) (Hao et al., 2015; Niu et al., 2016), in a manner similar to the repression of *FT* by the flowering inhibiting gene, *SVP*, in *Arabidopsis* (Lee et al., 2007). Similarly, expression of *FT1* in aspen was reduced by the overexpression of *SVL*, and enhanced by silencing *SVL* (Singh et al., 2018). The *CBF* genes can be quickly activated by cold stress, and in turn trigger many downstream genes involving growth cessation, cold acclimation and dormancy (Kendall et al., 2011). Overexpression of *CBF* was found to upregulate two *DAM* genes (*MdDAM1* and *MdDAM3*) in bud tissues of apple (Wisniewski et al., 2015). In a transient transformation and luciferase assay, Saito et al. (2015) demonstrated that CBF2 protein from Japanese pear (PpCBF2) regulates *DAM* expression through binding to the C-Repeat/Drought Response Element (CRT/DRE) motifs on the *DAM* genes. Confirmation of the direct regulation of *DAM* genes by CBF in Japanese pear (*P. pyrifolia*) (Niu et al., 2016; Li et al., 2019) and Japanese apricot (*P. mume*) (Zhang et al., 2018) prompted the hypothesis of a cold dependent CBF-DAM-FT model, in which cold temperature activates *CBF*, and CBF directly activates *DAM* genes, which repress the *FT* to arrest plant growth and result in the establishment of dormancy (Niu et al., 2016) (**Figure 2**).

### *DAM* and *SVL* Genes and Plant Hormones

Increasing evidence indicates the close interaction between *DAM/SVL* genes and plant hormones, especially ABA, suggesting that these hormones may be integral components in the *DAM/SVL* regulation cascade. For example, overexpression of *DAM6* (35S:PmDAM6) in Japanese apricot induced high accumulation of ABA and decreased CK contents in the terminal buds (Yamane et al., 2019). Transient assay and electrophoretic mobility shift assay (EMSA) indicate the *DAM*-induced ABA accumulation may be due to the binding of DAM to the CArG motif in the promoter of the ABA biosynthesis enzyme *NCED* (Tuan et al., 2017). This notion was supported by Singh et al. (2018)’s finding, in which *NCED3* was highly expressed in the transgenic aspen line with overexpression of *SVL*. On the other hand, *DAM/SVL* also mediates ABA signaling pathway. Using chromatin immunoprecipitation and ChIP-seq technique, Wu et al. (2018a) demonstrated that the *SVP2* from kiwifruit, which suppresses meristem activity and suppress bud break, can bind to the intron regions of *ABA insensitive ring protein 2*, and modulate the downstream genes. In aspen, overexpression of *SVL* positively regulated ABA receptor genes (*RCAR/PYL1* and *RCAR/PYL2*) genes (Singh et al., 2018). While *DAM* modulates ABA biosynthesis and signaling, ABA is also capable of regulating the expression of *DAM/SVL*. Early study in *Arabidopsis* reported that ABA can induce the expression of *CBF*, a positive regulator of *DAM* genes, *via* the CRT promoter element (Knight et al., 2004). Recent investigation in aspen showed that ABA application induces *SVL* expression and a mutation with reduced ABA response remarkably reduces *SVL* expression (Singh et al., 2018). Thus, ABA and *DAM/SVL* form a positive feedback loop in mediating dormancy. However, this notion was challenged by Tuan et al. (2017)’s finding in which an ABA singling gene *AREB1* represses the expression of *PpDAM1* by binding to the ABRE motifs in its regulatory region, indicating existence of a negative feedback regulation of ABA on *DAM*. To reconcile this contradiction, it is hypothesized that the ABA and *DAMs* interaction is dependent on the stage of dormancy: during dormancy induction, ABA promotes *DAMs* expression through activating *CBF*, which is also induced by low temperatures and SD, and as dormancy progresses, ABA inhibits *DAMs* activity through *AREB*-mediated negative feedback mechanism, and the reduced expression of *DAMs* at this stage should in turn repress ABA synthesis by downregulating *NCED* (**Figure 1**). In addition to ABA, *SVL* was also found to negatively affect GA as the GA biosynthesis gene *GA20ox* was reduced by the overexpression of *SVL*, but enhanced by its gene silencing after low temperature treatment (Singh et al., 2018). The finding of the versatility of *SVL* in targeting multiple dormancy-related genes raised the hypnosis that *SVL* may function as a hub gene that dictates both dormancy establishment and release by connecting ABA and GA, and cold perception pathways (Busov, 2019).

### Epigenetic Regulation of *DAM* and *SVL* Genes

In addition to the environmental and hormonal regulation, epigenetic modification has also been found to play an important role in regulating *DAM* expression during the dormancy phase transition. In the chromatin modification, histone H3 trimethylation at K4 (H3K4me3) and H3 acetylation (H3ac) are associated with activation of the nearby genes, whereas histone trimethylation at K27 (H3K27me3) represses transcription (Shilatifard, 2006). Concomitant with cold accumulation, several chromatin regions including the promoter of *DAM6* genes in peach are marked by the enrichment of H3K27me3 and removal of H3K4me3 and H3ac (Leida et al., 2012). In Japanese pear, Saito et al. (2015) showed that the reduction of expression of *DAM* homolog (*PpMADS13-1*) towards the dormancy release can be attributed to the decrease of H3K4me3, but not H3K27me3, which remained largely unaltered. Similarly, Singh et al. (2018) observed no significant increase of H3K27me3 at the *SVL* locus in low-temperature treated aspen. It was reported that H3K27me3 was specific to peach cultivars with high chilling requirement (Leida et al., 2012), and thus it appears that downregulation of *SVL/DAM* genes may be achieved predominantly *via* the reduction of H3K4me3, whereas H3K27me3 modification is reserved for species or cultivars that require high chill accumulation. This histone modification in *DAM* genes is highly reminiscent of that in *FLC* locus in *Arabidopsis* during winter vernalization (Bastow et al., 2004; He et al., 2004). In sweet cherry, chill accumulation leads to DNA methylation in the promoter of DAM genes and increase of the small interfering RNAs (*siRNA*) that match this region, concomitant with the upregulation of *FT* (Rothkegel et al., 2017). Similar *DAM* genes degradation mediated by siRNA (*miR6390*) and the subsequent release of FT2 was also found in pear (Niu et al., 2016). These findings indicate the epigenetic modification is a main mechanism that regulates *DAM* genes and highlight the involvement and importance of *DAM* genes during dormancy.

## Final Remarks and Perspectives

Although many plant hormones are implicated in dormancy regulation, some aspects are still open to questions. ABA is the central regulator of dormancy, and its repression of cell cycle and intercellular communication *via* plasmodesmata appears to be important mechanisms leading to dormancy induction. The role of GA is largely within its conventionally defined function: promotion of cell division and elongation, which is an essential step in dormancy release. However, whether GA directly unlock dormancy still needs further investigations. ET acts antagonistically with GA during dormancy induction, but the byproduct of its metabolism seems able to promote dormancy release. The direct interaction between *DAM/SVL* genes and ABA opens the door to hypothesize that *DAM/SVL* may regulate dormancy through modifying the metabolism and/or signaling of other hormones, such as GA and CK biosynthesis. The interaction between *DAM/SVL* genes and other plant hormones is expected to be further revealed in the coming years. The potential roles of *DAM* and *SVL* in the mediation of dormancy, though seemingly distinct, are expected to be integrated in a broader context of an overarching theme of growth inhibition, rather than dormancy *per se*. Thanks to the fast-growing research on bud dormancy in recent years, much have been learned about the hormone induction and repression kinetics during dormancy, the role of hormone biosynthesis and signaling-related genes, effects of hormones and their cross talks during the initiation, progression and release of bud dormancy. However, the fundamental molecular and cellular mechanisms that initiate the transitions between meristematic growth, bud arrest, dormancy and resumption of metabolic activities still require further elucidation.

## Author Contributions

JL and SS have contributed equally to the writing, editing and preparation of this review article.

## Funding

The authors are grateful for the funding to SS from The Virginia Agricultural Council (#449846) and The Virginia Apple Research Program (#459952).

## Conflict of Interest Statement

The authors declare that the research was conducted in the absence of any commercial or financial relationships that could be construed as a potential conflict of interest.

## Abbreviation

ABA, abscisic acid; CALS1, callose synthase; CDK, cyclin-dependent kinases; CK, cytokinin; *DAM*, dormancy-associated MADS genes; ET, ethylene; FT, flowering locus T; GA, gibberellins; GA2ox, GA 2-oxidase; GA3ox, 3-oxidase; GA20ox, dioxygenases 20-oxidase; HC, hydrogen cyanamide; IAA, indole-3-acetic acid; JA, jasmonate; NCED, 9-*cis*-epoxycarotenoid dioxygenase; PP2Cs, protein phosphate 2Cs; ROS, reactive oxygen species; SD, short day; SVP, short vegetative phase.
